# Case Report: A Paternal 20q13.2-q13.32 Deletion Patient With Growth Retardation Improved by Growth Hormone

**DOI:** 10.3389/fgene.2022.859185

**Published:** 2022-03-24

**Authors:** Yu Liu, Ying Yang, Liming Chu, Shuai Ren, Ying Li, Aimin Gao, Jing Wen, Wanling Deng, Yan Lu, Lingyin Kong, Bo Liang, Xiaoshan Shao

**Affiliations:** ^1^ Department of Pediatric Endocrinology, Genetics and Metabolism, Guiyang Maternal and Child Health Care Hospital, Guiyang Children’s Hospital, Guiyang, China; ^2^ Basecare Medical Device Co., Ltd., Suzhou, China; ^3^ State Key Laboratory of Microbial Metabolism, Joint International Research Laboratory of Metabolic and Developmental Sciences, School of Life Sciences and Biotechnology, Shanghai Jiao Tong University, Shanghai, China; ^4^ Department of Renal Rheumatology and Immunology, Guiyang Maternal and Child Health Care Hospital, Guiyang Children’s Hospital, Guiyang, China

**Keywords:** short stature, growth retardation, 20q13.2-13.32, growth hormone, gnas

## Abstract

Interstitial chromosome 20q deletions, containing *GNAS* imprinted locus, are rarely reported in the past. Hereby, we presented a Chinese boy with a novel 4.36 Mb deletion at paternal 20q13.2-13.32, showing feeding difficulty, malnutrition, short stature, lower limb asymmetry, sightly abnormal facial appearance and mild intellectual abnormality. With 3 years’ growth hormone treatment, his height was increased from 90 to 113.5 cm. This report is the first time to describe the outcome of clinical treatment on a patient with this rare chromosomal 20 long arm interstitial deletion, containing *GNAS* locus, which may facilitate the diagnosis and treatment of this type of patient in the future.

## Introduction

The most commonly observed chromosome 20 abnormality is a ring-shaped chromosome 20, r (20). Those patients with r (20) are characterized by growth retardation, intellectual problems, facial dysmorphism, seizures and specific electroencephalogram pattern (Porfirio et al., 1987). However, interstitial chromosomal deletions around 20q13.2-13.32, containing *GNAS* locus, are very rare. Till now, only 7 patients from 5 reports were shown to possess this type of deletions. They all shared similar phenotypes of feeding difficulty, growth retardation, abnormal facial characteristics, abnormal subcutaneous fat distribution and intellectual disability (Aldred et al., 2002; Geneviève et al., 2005; Butler et al., 132013; Balasubramanian et al., 2015; Solomon et al., 2011). Here, we detected a unique 4.36 Mb paternal deletion on chromosome 20q13.2-q13.32 in a Chinese boy *via* next generation sequencing (NGS)-based CNV analysis and SNP array-based haplotyping. He showed common phenotypes compared with the other patients. Remarkably, he had good response to growth hormone (GH) treatment, and his height was increased by 23.5 cm after about 3 years’ treatment.

## Materials and Methods

### Patient’s Informed Consent and Sample Collection

All genetic tests were based on peripheral blood samples collected from the patient and his parents. Informed consent was obtained from the patient and his parents for this study in accordance with the Helsinki principles for enrollment in research protocols approved by the Ethics Committee of Guiyang Maternal and Child Health Care Hospital, Guiyang Children’s Hospital.

### Whole Genome Copy Number Variation Analysis

Genomic DNA (gDNA) was extracted from blood leukocytes of the patient and his parents using TIANamp Blood DNA kit (TIANGEN). CNVs analysis was first performed *via* an NGS-based low depth whole genome sequencing. The data was then interpreted by TMAP (Version 4.6), Picard (Version 2.18.17), and the method of LOWESS regression and circular binary segmentation were applied to analyze CNV (Xia et al., 2019).

### Haplotyping *via* SNP Array

Illumina Infinium Asian Screening Array-24 v1.0 was used to identify the SNPs in the whole genome and the SNPs were adopted to haplotype linkage analysis according to the instructions in a previous paper (Zhang et al., 2017).

## Results

### Patient Characteristics and Clinical Observation

**FIGURE 1:**
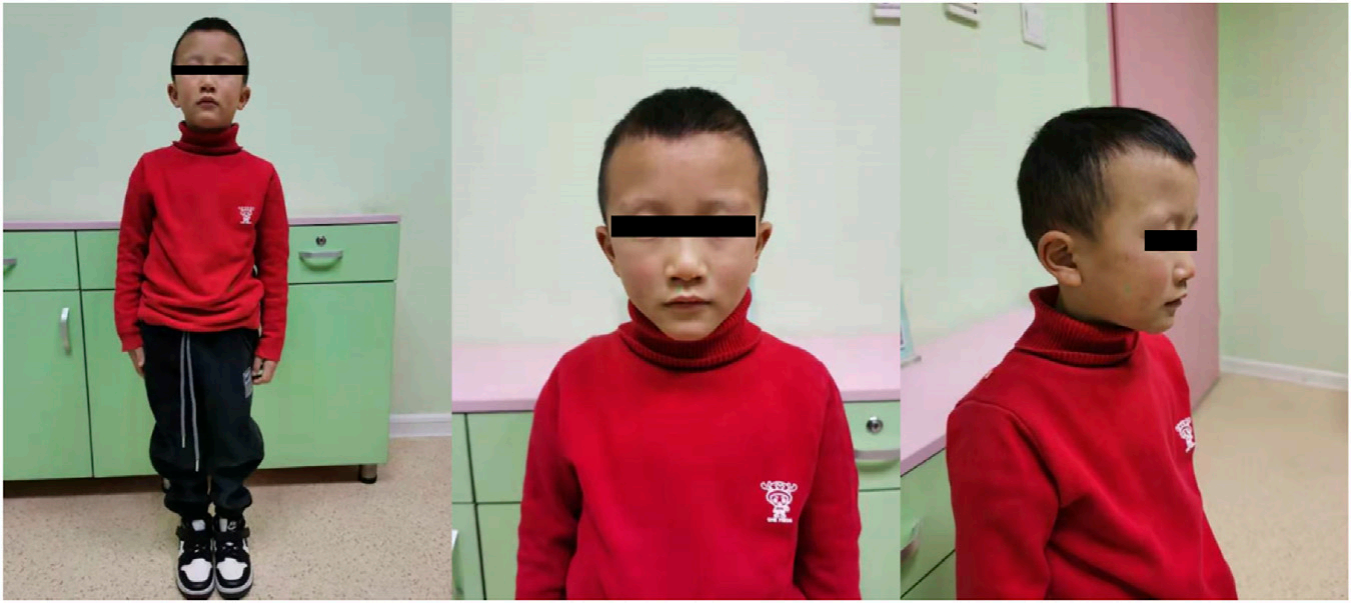
Front and side view of the patient. The patient had a triangular face, small and pointed chin and sparse-hair eyebrows, and shortened left lower limb.

The patient in this case was the second child born from healthy Chinese parents, and his elder brother showed normal phenotype. The affected boy experienced intrauterine asphyxia during gestation and was born *via* caesarean section after full term of gestation. Birth measurements showed he was small for gestational age (SGA) (length = 47 cm; weight = 1.92 kg) and exhibited talipes equinus, so he accepted orthosis at one-year-old, lasting more than 6 months. According to his parents’ description, this patient experienced severe feeding difficulty after he was born. He also suffered from malnutrition, shortened left lower limb and small bilateral testes. Moreover, he had mild learning difficulties and achieved very poor grades in a mainstream class. In terms of his facial features, he had a triangular face, small and pointed chin and sparse-hair eyebrows (Figure 1) He first came to the clinic at 5 years of age due to severe postnatal growth retardation (height = 90 cm, <-3SD; weight = 10 kg). The patient’s pituitary hormone test exhibited increased level of thyroid-stimulating hormone (TSH: 5.85 μIU/ml), reduced but normal level of insulin-like growth factor-1 (IGF1: 3.25 nmol/L) and normal growth hormone (GH) release (peak GH level in L-dopa test = 24.5 ng/ml; peak GH level in Arginine test = 5.12 ng/ml).

### Clinical Treatment and Follow up

**FIGURE 2:**
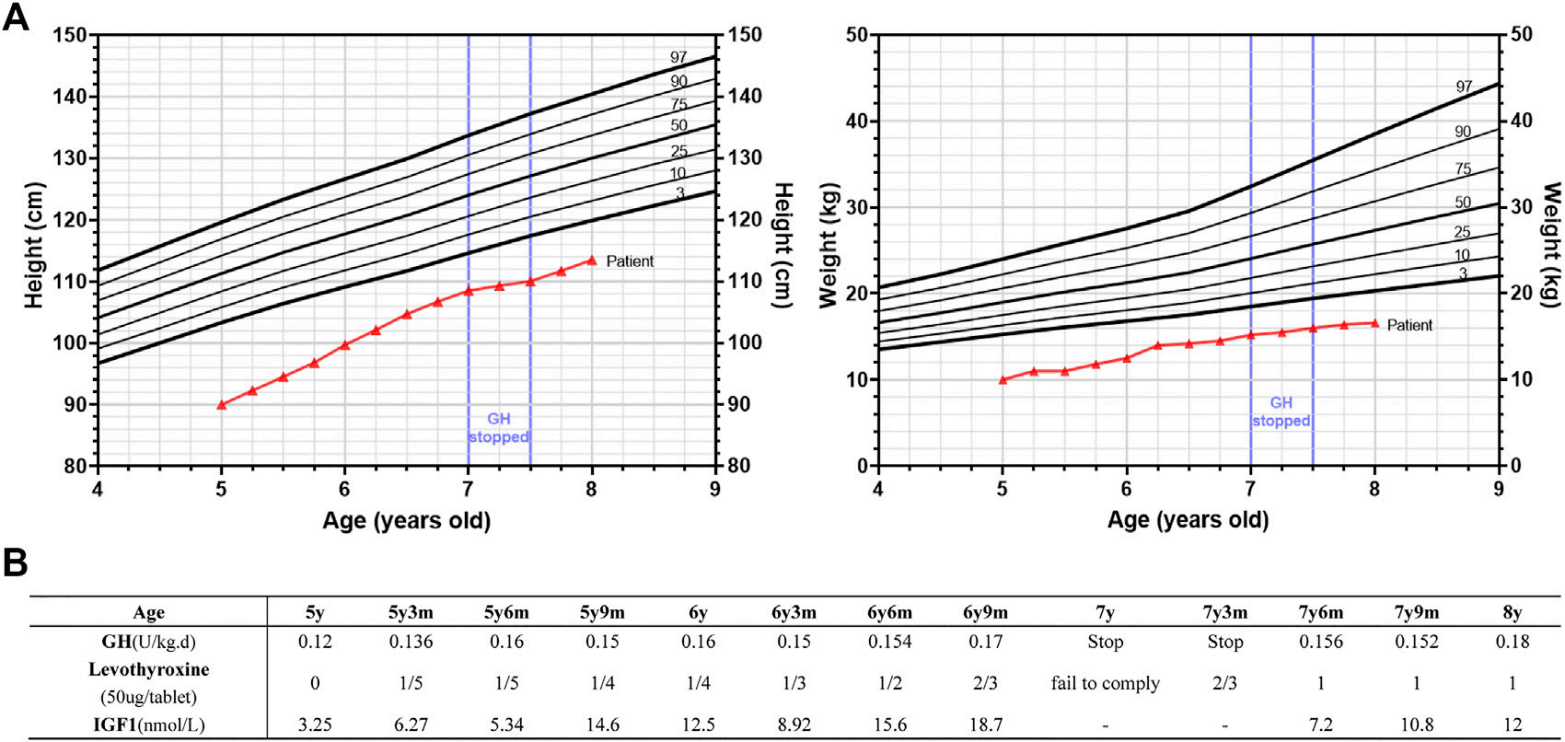
Growth of the patient’s height and weight during growth hormone (GH) and adjuvant levothyroxine treatment. **(A)** Height and weight standard deviation curve of Chinese 4–9 years old boys and the growth trend of the height (left panel) and weight (right panel) of the patient from 5y (years old) to 8y. The growth curve of the patient was indicated in red lines. GH treatment was suspended from 7y to 7y6m (months) as indicated. Different percentiles (3rd, 10th, 25th, 50th, 75th, 90th, and 97th) were as indicated. **(B)** The dosage of GH and levothyroxine used, and the corresponding IGF1 levels during this period. GH treatment was suspended from 7y to 7y6m (months) and levothyroxine was added as an adjuvant therapy when TSH reached 7.77 μIU/ml after 3 months of GH treatment.

After the initial attendance to the clinic, GH treatment was given to him for 2 years. Levothyroxine was added as an adjuvant therapy when TSH reached 7.77 μIU/ml after 3 months of GH treatment. His height was increased to 108.5 cm at 7 years old (about 9.25 cm/year, from 5–7 years old), and the TSH level was decreased to 4.98 μIU/ml. However, the patient’s family refused to continue using GH due to financial concerns at that time, so the boy only gained 1.5 cm in the following 6 months. After restoring the GH treatment, his growth rate increased again (3.5 cm/6 months), which means GH indeed promoted the growth of the patient, although he was still much shorter than the average (-3SD) (Figure 2). Thus, the patient were 90 cm (-4.93SD, < 3rd percentile, 5 years old) before GH treatment, 99.7 cm (-3.87SD, < 3rd percentile, 6 years old), 108.5 cm (-3.06SD, < 3rd percentile, 7 years old) and 113.5 cm (-3.03SD, < 3rd percentile, 8 years old) after GH treatment. The IGF1 levels were within normal range during GH treatment (Figure 2B). The patient was malnourished to some degree and we just advised him to increase his nutrition in the diet without special treatment.

### Genetic Analysis in the Patient and his Parents

**FIGURE 3:**
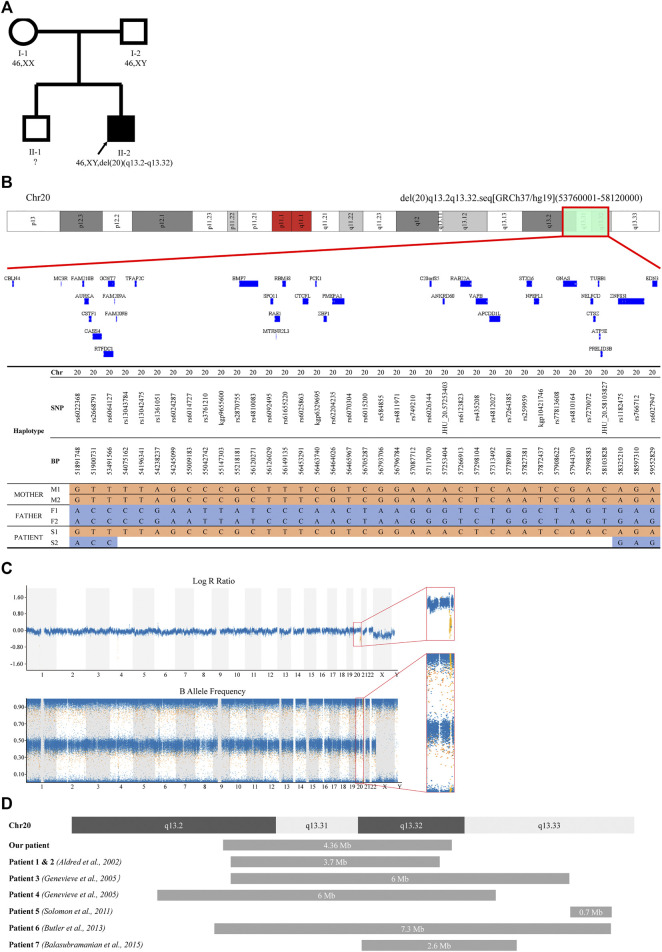
Pedigree and CNV analysis of the family. **(A)** Familial pedigree showing the phenotype and genotype of the family. Proband (II-2) had a heterozygous deletion of chromosome 20q13.2-q13.32. The genotype of proband’s elder brother (II-1) was not assessed. **(B)** A schematic structure of the deleted regions detected in the patient (II-2) and comparison with his parents (I-1 and I-2). Only SNP positions at which both parents were homozygous for opposite alleles were showed. **(C)** B allele frequency plot and log R ratio plot of the patient’s SNP array result. **(D)** A schematic diagram of reported deletions around 20q13.32 compared with the deletion identified in this study.

In contrast to the parents, who were healthy and had normal height (father’s height = 175 cm; mother’s height = 160 cm), the patient himself showed short stature, intellectual abnormality and subclinical hypothyroidism (Figure 3A). There was also no consanguinity between the parents. We hypothesized the patient may suffer from an autosomal recessive disease that was inherited from his parents, or an autosomal dominant disease that resulted from a *de novo* mutation. Thus, whole exome sequencing (WES) and whole genome copy number variation (CNV) analysis were carried out in this patient and his parents. WES did not reveal any relevant pathogenic variants in the family, while CNV analysis showed the presence of 46,XY,del (20) (q13.2-q13.32) in the patient’s genome (Supplementary Figure S1A). This deletion was about 4.36 Mb in size. However, the parents’ genomes were shown to be intact. Further comparison between the proband and his parents’ haplotypes confirmed his paternal copy of chromosome 20q13.2-q13.32 was lost (Figures 3B,C). Since the patient’s brother and both parents showed normal phenotype, and the parents both showed no such deletion in chromosome 20 (Supplementary Figure S1B), so this rare deletion was implied to be a *de novo* mutation.

## Discussion

In this case report, we presented a Chinese boy showing learning difficulties, growth retardation and endocrine abnormalities, with a rare *de novo* 4.36 Mb deletion on paternal 20q13.2-q13.32. Via exploring the online database (http://grch37.ensembl.org/index.html), this region was found consisting of 35 protein coding genes and 27 non-coding genes. Among them, there are 9 OMIM genes (*MC3R*, *AURKA*, *PCK1*, *VAPB*, *TUBB1*, *STX16*, *ATP5E*, *GNAS* and *EDN3*). Noteworthy, *GNAS* is a haploinsufficient gene locus highly associated with his phenotype (Turan and Bastepe, 2013).

*GNAS* is an important imprinted locus that is responsible for the production of multiple transcripts: paternally expressed GNAS-A/B, GNAS-AS1, GNAS-XLαs and maternally expressed NESP55, while Gαs is biallelically expressed in most tissues, with maternal expression in some specific tissues (eg. neonatal brown tissue, gonads and thyroids) (Bastepe, 2007; Turan and Bastepe, 2015). Gsα is the most well-studied transcript expressed from *GNAS* locus, encoding the alpha subunit of G-protein involving in guanine nucleotide-binding. G-protein can trigger complex signaling networks *via* GDP-GTP exchange. The activated form of GTP-bound Gsα can stimulate different effectors, such as adenylyl cyclase, and thereby initiate various cellular responses, including in endocrine glands (Cabrera-Vera et al., 2003; Bastepe and Jüppner, 2005; Turan and Bastepe, 2015). For example, adenylyl cyclase facilitates the production of cAMP, which can bind to the protein kinase A (PKA) and further lead to the activation of its catalytic subunit. Catalytic-active PKA is involved in the iodine uptake and the expression of genes associated with thyroid hormone production (Pereira et al., 2020). As a result, mutation of *GNAS* can cause different types of endocrine-related diseases, such as McCune-Albright syndrome (OMIM#174800), progressive osseous heteroplasia (OMIM#166350) and pseudohypoparathyroidism (PHP).

It is worth to note that different forms of PHP can be triggered by heterozygous mutation of *GNAS*, such as PHP-Ia, PHP-1b, and pseudopseudohypoparathyroidism (PPHP) (Mantovani et al., 2020). PHP-Ia and PPHP are caused by inactivating mutations in Gs-a, while PHP-1b is due to an abnormal methylation on *GNAS* imprinted regions (Turan and Bastepe, 2015; Mantovani et al., 2020). PHP-Ia patients are characterized by short stature, subcutaneous ossification, brachydactyly and variable degrees of intellectual disability, so called Albright’s Hereditary Osteodystrophy (AHO), as well as parathyroid hormone (PTH) resistance. It may also show resistance to other hormones, such as growth hormone releasing hormone (GHRH) and TSH (Aldred et al., 2000; Weinstein et al., 2001; Levine, 2002). PPHP patients show most AHO phenotypes, but not presenting hormone resistance (Aldred et al., 2000; Mantovani et al., 2020). PPHP often develops when the paternal Gs-a is deficient, while PHP-Ia develops when the maternal one is inactivated (Davies and Hughes, 1993). The less severe phenotype of PPHP may be due to the tissue specific paternal imprint of Gs-a. In our case, the phenotype and genotype of our patient is more consistent with the PPHP. However, deletion of other genes may also play roles in the resulting phenotypes.

To date, there were only 7 patients reported to carry this rare deletion around 20q13.32 containing *GNAS* imprinted locus (Figure 3D), and 4 of them were confirmed to have paternal deletion (Table 1). Almost all of those affected by 20q13.32 deletion containing *GNAS* showed reduced birth weight, abnormal facial appearance, feeding difficulties, intellectual disabilities and growth retardation (Aldred et al., 2002; Geneviève et al., 2005; Butler et al., 132013; Balasubramanian et al., 2015; Solomon et al., 2011). The two cases reported by Aldred et al. showed obesity, and those reported by Genevieve et al. had uneven subcutaneous fat distribution, while the patient reported by Balasubramanian et al. had reduced adipose tissue (Aldred et al., 2002; Geneviève et al., 2005; Balasubramanian et al., 2015). Compared with those previous reports, the patient in our case showed similar triangular face, sparse-hair eyebrows, low birth weight, feeding problems, reduced adipose tissue, severe growth delay and intellectual abnormalities. However, when compared with UPD(20)mat and Silver Russell Syndrome (SRS)-like phenotypes, the clinical manifestations in our patient (paternal 20q13.2-q13.32 deletion) was a little bit different. As previously reported, patients with UPD(20)mat generally have phenotypic features such as growth retardation, including intrauterine growth retardation (IUGR), feeding difficulties, about 1/5 patients shared phenotypes like: hypotonia, dysmorphic features, developmental delay (DD) and Silver-Russel-syndrome like phenotypes [http://cs-tl.de/DB/CA/UPD/0-Start.html (accessed 02/25/2022)]. And the clinical diagnosis of SRS was based on six features: pre- and postnatal growth failure, relative macrocephaly, prominent forehead, body asymmetry, and feeding difficulties (Netchine–Harbison clinical scoring system (NH-CSS)) (Tannorella et al., 2021). So our patient shared growth retardation and feeding difficulties with UPD(20)mat patients, and shortened left lower limb fits the phenotype of Silver Russell Syndrome, 3/6 met the NH-CSS criteria. Accordingly, phenotypes of this 20q deletions were different from that of UPD(20)mat and SRS, mainly in the triangular face, sparse-hair eyebrows, reduced adipose tissue, and intellectual abnormalities. In mice and humans, loss of function of XLαs was associated with intrauterine and perinatal growth retardation, as well as poor adaption to feeding (He et al., 2015), which was similar to our patient. But XLαs deficiency also caused hypoglycemia and disrupted glucose counter-regulation which did not occur in our patient (He et al., 2015). Considering that the paternal GNAS allele was deleted, it was also predicted that the patient might have some clinical features of Albright hereditary osteodystrophy (AHO). In our case, The patient had part of the characteristics of AHO, such as: short stature, brachydactyly, he also had limb asymmetry, but he did not have other characteristics of AHO such as obesity, round face and subcutaneous ossification.

**TABLE 1:** Clinical features of patients with deletion around 20q13.32 containing *GNAS* locus.

	Patient 1	Patient 2	Patient 3	Patient 4	Patient 5	Patient 6	Patient 7	Patient 8
	Aldred et al., 2002		Genevieve et al., 2005		Solomon et al., 2011	Butler et al., 2013	Balasubramanian et al., 2015	Our Patient
Ethnicity	Unknown	Turkish	Caucasian	Caucasian	Caucasian	Peruvian	Caucasian	Chinese
Deleted region	20q13.13-13.32	20q13.31-13.33	20q13.2-13.3	20q13.2-13.3	20q13.33	20q13.2-13.33	20q13.32-13.33	20q13.2-13.32
Origin of deletion	Paternal	Maternal	Paternal	Paternal	*de novo*	Unknown	Paternal	Paternal
Gender	Male	Female	Female	Female	Male	Female	Male	Male
Birth weight (kg)	1.12	Unknown	1.32	1.57	Unknown	1.47	1.56	1.92
Feeding difficulty	Yes	Unknown	Yes	Yes	Unknown	Yes	Yes	Yes
Facial features	Elongated face, broad tip of nose, sparse hair lateral eyebrows, prominent chin, large ears with wide antihelix	Unknown	High forehead, floppy and low-set ears, enophthalmia and dysplastic iris, flat broad nasal bridge, short and prominent philtrum, small chin	High forehead, floppy and low-set ears, enophthalmia and dysplastic iris, flat broad nasal bridge, short and prominent philtrum, small chin	Unknown	Triangular face with open mouth, low-set ears, hypertelorism and ptosis eyes, flat broad nasal bridge, unilateral cleft lip, small and pointed chin	Triangular face, low-set ears, grayish sclerae, pointed chin	Triangular face, pointed chin, sparse-hair eyebrows
Talipes equinovarus	Yes	Unknown	No	No	Unknown	No	No	Yes
Bone age	Delayed	Unknown	Delayed	Delayed	Unknown	Delayed	Delayed	Delayed
Subcutaneous fat distribution	Obese	Obese	Uneven	Uneven	Unknown	Unknown	Reduced adipose tissue	Reduced adipose tissue
Intellectual disability	Mild	Mild	Moderate-severe	Moderate	Unknown	Moderate	Mild	Mild
Growth retardation	Yes	Yes	Yes	Yes	Yes	Yes	Yes	Yes

The dosage of GH was based on the guideline recommendations for the clinical use of recombinant human growth hormone in Chinese children: childhood GHD: 0.075- 0.15 U/(kg d), ISS: 0.125-0.2 U/(kg d) (Subspecialty Group of End, 2013). The amount of GH used was in this range (Figure 2B). Considering the initial age (5 years) and bone age of the child, and the space for subsequent height gain is still relatively large, our initial GH dose is slightly smaller (0.12 U/(kg d) at 5 years old), and then gradually increased to 0.18 U/(kg d) (at 8 years old) because the growth rate is not very satisfactory. A recent study showed that, after 1 year of GH treatment, HtSDS (Height standard deviation score) increased in 13 ISS children with normal IGF1 level (start at -2.06, end at -1.76) and 17 GHD children with normal IGF1 level (start at -2.23, end at -1.87) (Soliman et al., 2021). GH therapy was relatively effective in SRS patients. Meropi Toumba et al. repoted that 26 SRS patients received GH treatment for at least 7 years [median duration of treatment was 9.8 years (range 7.0-15.7)], and the median height of the 26 patients at the commencement of treatment increased significantly by the end of treatment (height SDS: start at -2.74, end at -1.33) (Toumba et al., 2010). As for GH treatment in PPHP patient, R Manfredi et al. repoted a female PPHP patient, who received a 3-yr-6 months GH treatment. As a result, a significant improvement of growth velocity was obtained, while the height SDS failed to improve (-3.30 pretreatment (10 years old); -3.14(11 years old), -3.36(12 years old) and -3.59 (13 years old) after the first, the second and the third year of treatment, respectively) (Manfredi et al., 1993). As for our patient, although he failed to return to normal height with GH treatment, his height SD changed more in the first 2 years (5–7 years old) than that in those reports (Figure 2A).

Although GH treatment did not restore the child’s height to normal, the increase of about 9.25 cm/year in height during GH treatment (GH treatment data from 5–7 years old) was higher than that of normal Chinese male children by 6.35 cm/year (5–7 years old) (Li et al., 2009), so GH treatment was beneficial for this child. If GH treatment was not taken at that time, the child’s height would be shorter, just as he suspended GH treatment between 7–7.5 years old, and the height increased by only 1.5 cm in half a year. The IGF1 level of 3.25 nmol/L (at initial diagnosis) and the change in IGF1 levels during GH therapy afterwards (Figure 2B), although low, were within the normal range (4–6 years: 2.86-27.04 nmol/L; 7–9 years: 5.2-33.15 nmol/L) (Xu et al., 2010).

Moreover, a genetic testing method combining NGS-based CNV analysis and SNP array-based haplotyping could become a powerful tool to precisely identify the location of a chromosomal deletion and trace its parent of origin. There are also some limitations to our research, due to the limitation of sample collection, the relationship between methylation status in this region and phenotype needs further study in the future.

In conclusion, here we presented a Chinese patient with a novel interstitial deletion around 20q13.2-13.32, containing *GNAS* locus. He exhibited high concordance with previously reported phenotypes. However, none of the previous reports presented any treatment information on the patients. Therefore, this case report was the first time to provide clinical treatment data on a patient with such rare interstitial deletion on chromosome 20, which shows GH treatment did have a positive effect on his growth.

## Data Availability

The datasets for this article are not publicly available due to concerns regarding participant/patient anonymity. Requests to access the datasets should be directed to the corresponding authors.
