# Supplementary figures and images for: Case Report: A Paternal 20q13.2-q13.32 Deletion Patient With Growth Retardation Improved by Growth Hormone
Source: Front Genet. 2022 Mar 24;13:859185. doi: 10.3389/fgene.2022.859185 (PMC8987769; doi:10.3389/fgene.2022.859185)

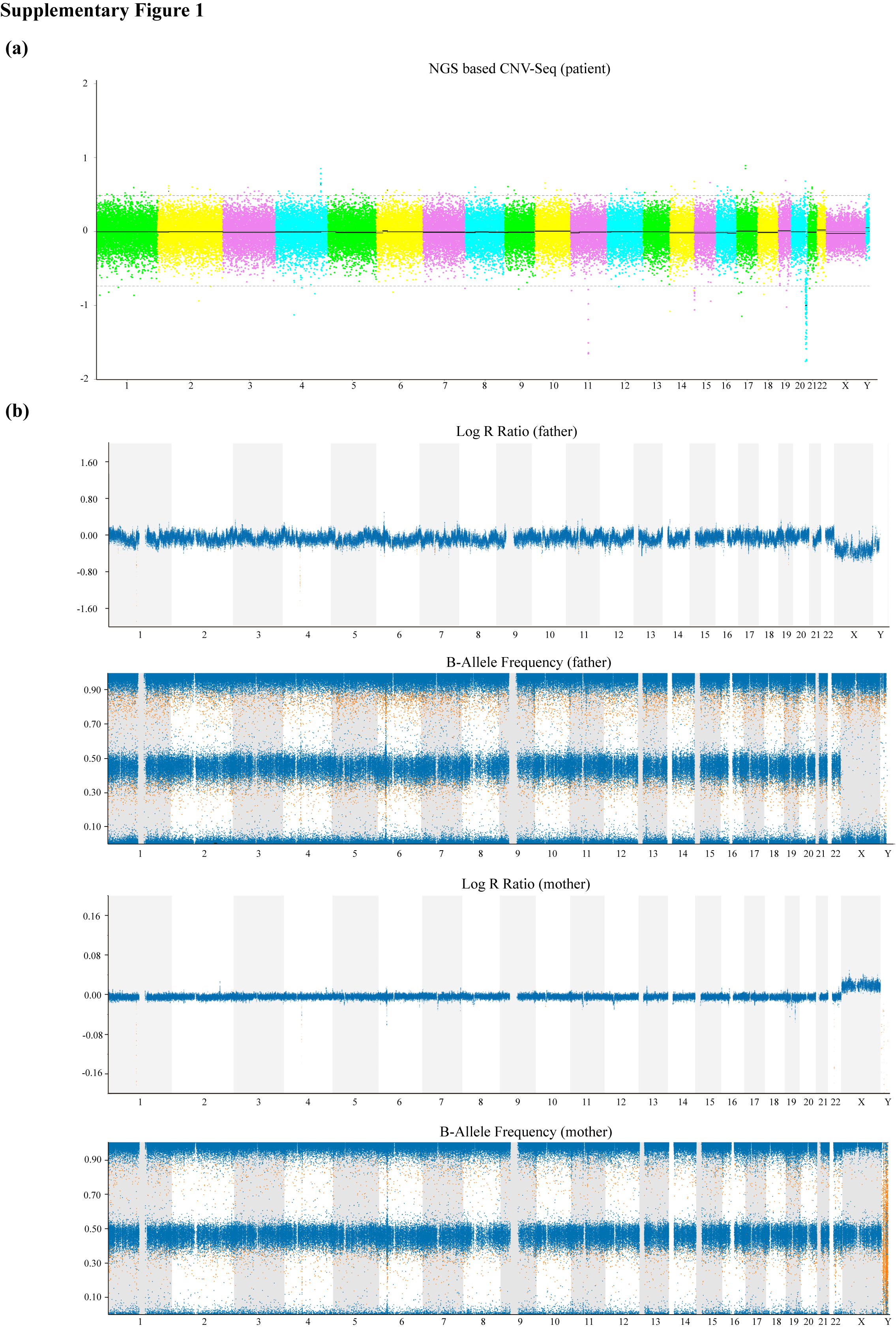

Supplement: Supplementary file 1 [file Image1.TIF]
